# Analysis of Personalized Cardiovascular Drug Therapy: From Monitoring Technologies to Data Integration and Future Perspectives

**DOI:** 10.3390/bios15030191

**Published:** 2025-03-17

**Authors:** Runxing Lin, Ziyu Huang, Yu Liu, Yinning Zhou

**Affiliations:** Joint Key Laboratory of the Ministry of Education, Institute of Applied Physics and Materials Engineering, University of Macau, Avenida da Universidade, Taipa 999078, Macau

**Keywords:** sensors, dynamic monitoring, multidimensional data integration and analysis, personalized cardiovascular drug therapy

## Abstract

Cardiovascular diseases have long been a major challenge to human health, and the treatment differences caused by individual variability remain unresolved. In recent years, personalized cardiovascular drug therapy has attracted widespread attention. This paper reviews the strategies for achieving personalized cardiovascular drug therapy through traditional dynamic monitoring and multidimensional data integration and analysis. It focuses on key technologies for dynamic monitoring, dynamic monitoring based on individual differences, and multidimensional data integration and analysis. By systematically reviewing the relevant literature, the main challenges in current research and the proposed potential directions for future studies were summarized.

## 1. Introduction

Cardiovascular diseases are a group of common chronic conditions that affect the function of the heart and blood vessels, including coronary heart disease [1], hypertension [2], and heart failure [3]. These diseases often lead to blood circulation disorders, which, in severe cases, may result in strokes [4] or even life-threatening situations [5]. Due to their high mortality and disability rates, cardiovascular diseases pose a significant burden on individual health and societal healthcare resources [6]. Comprehensive management of cardiovascular diseases, from prevention to follow-up, is crucial, as it not only helps reduce incidence rates but also improves patients’ long-term prognosis [7]. In recent years, cardiovascular diseases have shown trends of steadily increasing incidence, a noticeable shift toward younger populations, a higher proportion of comorbidities with other chronic diseases, and insufficient treatment adherence [8,9,10]. These factors further complicate this public health issue. Against this backdrop, the prevention and treatment of cardiovascular diseases are of paramount importance [11,12].

Pharmacological treatment of cardiovascular diseases has achieved remarkable success in reducing mortality and improving patient prognosis. However, its complexity and challenges remain prominent. This complexity spans various stages, including disease diagnosis, treatment, and efficacy monitoring, further underscoring the necessity and importance of personalized treatment [13]. In terms of diagnosis, the etiology and clinical manifestations of cardiovascular diseases are highly complex, and some patients may present with atypical symptoms, which poses significant challenges to early diagnosis [14,15]. In terms of treatment, pharmacological therapy is one of the most common interventions. However, due to the narrow therapeutic window of certain drugs, even slight deviations in dosage can lead to severe consequences, imposing stringent requirements on treatment precision. Consequently, frequent efficacy monitoring and treatment adjustments based on monitoring results become essential [16,17].

Nevertheless, drug efficacy is often influenced by multiple factors, such as the patient’s genetic background, metabolic capacity, and underlying comorbidities [18,19,20]. These factors result in significant variability in the sensitivity and efficacy of the same medication among different patients. While some patients may experience significant therapeutic benefits, others may encounter insufficient efficacy or severe adverse reactions [21]. Furthermore, for cardiovascular disease patients with comorbidities (e.g., diabetes), combination therapy is a common treatment strategy. However, drug–drug interactions may reduce efficacy or exacerbate adverse effects, adding further complexity to treatment [22]. At the same time, traditional efficacy monitoring methods also have certain limitations. For example, some patients may have a fear of or discomfort with blood sampling, especially those sensitive to needles. Frequent invasive blood sampling for monitoring may reduce patient adherence [23,24]. Therefore, for medications requiring real-time dynamic dose adjustment, the intermittent monitoring methods widely used in clinical practice (e.g., periodic blood tests) may be inadequate for capturing real-time changes in drug concentrations and efficacy. For example, adjusting the dosage of anticoagulant medications typically relies on monitoring the international normalized ratio (INR). Generally, in the treatment of venous thromboembolism, INR is monitored daily during the initial phase of treatment, and the warfarin dosage is adjusted accordingly. Once INR stabilizes, the monitoring interval can be extended to a maximum of 12 weeks after three months [25]. Medications like warfarin require precise dosage adjustments but are easily influenced by factors such as diet, other medications, and genetic factors. Although experts recommend that patients’ time in therapeutic range (TTR) should reach at least 65%, this is rarely achieved in practice [26]. This delay in monitoring restricts the ability to dynamically adjust treatment plans, potentially resulting in therapeutic adjustments lagging behind the patient’s actual needs [26,27,28,29]. A study has shown that patients who perform weekly self-testing (PST) have significantly higher TTR compared to those who test irregularly (every 2–4 weeks) (74% vs. 68.9%, *p* < 0.0001), with a lower incidence of extreme INR values [30]. This indicates that timely and frequent monitoring is more conducive to INR adjustment.

**Figure 1:**
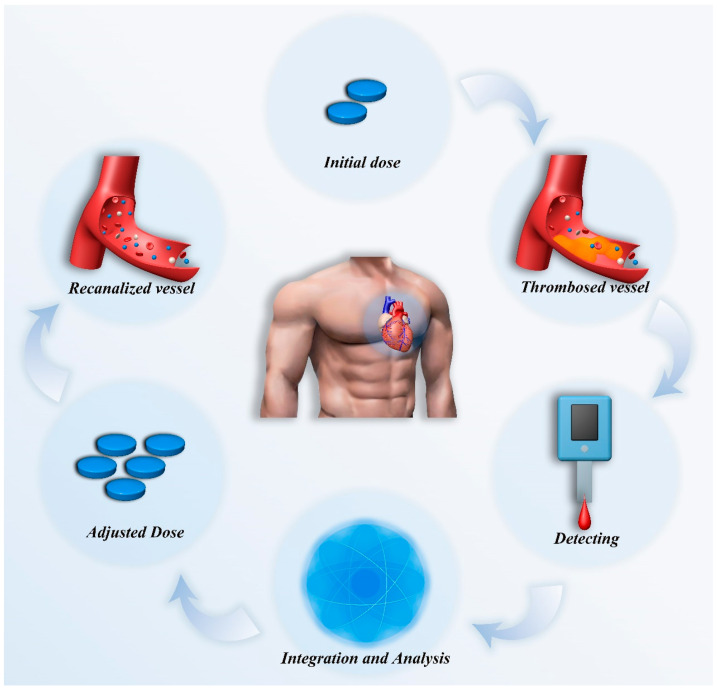
Schematic diagram of dynamic monitoring combined with multidimensional data analysis for adjusting medication in thrombotic patients as an example.

In summary, the complexity of pharmacological treatment, the challenges in diagnosis, and the limitations of traditional monitoring methods in cardiovascular disease management all highlight the core value of personalized treatment [31,32]. By dynamically monitoring patients’ physiological biomarkers and related data, and integrating multidimensional data analysis to enable dose adjustments, personalized treatment offers a crucial pathway forward (Figure 1). It allows for the development of optimized treatment plans tailored to the specific conditions of each patient, thereby improving efficacy, reducing adverse reactions, and enhancing long-term prognosis. This represents not only a key direction in the development of cardiovascular disease treatment but also a critical breakthrough path in addressing current complexities and challenges [32,33]. This review will systematically elaborate on the current status and prospects of dynamic monitoring in cardiovascular pharmacological treatment from the following four aspects: key technologies for dynamic monitoring, dynamic monitoring based on individual differences, multidimensional data integration and analysis, as well as challenges and future developments. This review aims to explore the critical pathways and strategies for achieving personalized pharmacological treatment, providing new insights and inspiration for the precise treatment of cardiovascular diseases.

## 2. Key Technologies for Dynamic Monitoring

Monitoring technologies play a crucial role in cardiovascular drug monitoring and efficacy evaluation. Especially in clinical applications, these technologies have been widely adopted and have had a profound impact [34,35]. At present, the key technologies for dynamic monitoring of cardiovascular drug efficacy include sensor technology, microfluidic chip technology, and the application of nanomaterials [36]. These technologies enable high-sensitivity and multi-parameter detection [37,38]. As technology continues to advance, these methods have not only improved monitoring efficiency but also significantly expanded their potential applications in personalized medicine. Continuous monitoring, real-time monitoring, flexibility, portability, accuracy, and sensitivity have gradually become key characteristics that enable monitoring technologies to play a crucial role in personalized cardiovascular treatment. Through these characteristics, monitoring technology can more comprehensively collect individualized patient information, thereby providing a solid foundation for accurate diagnosis and the scientific development of personalized treatment plans [39].

**Figure 2:**
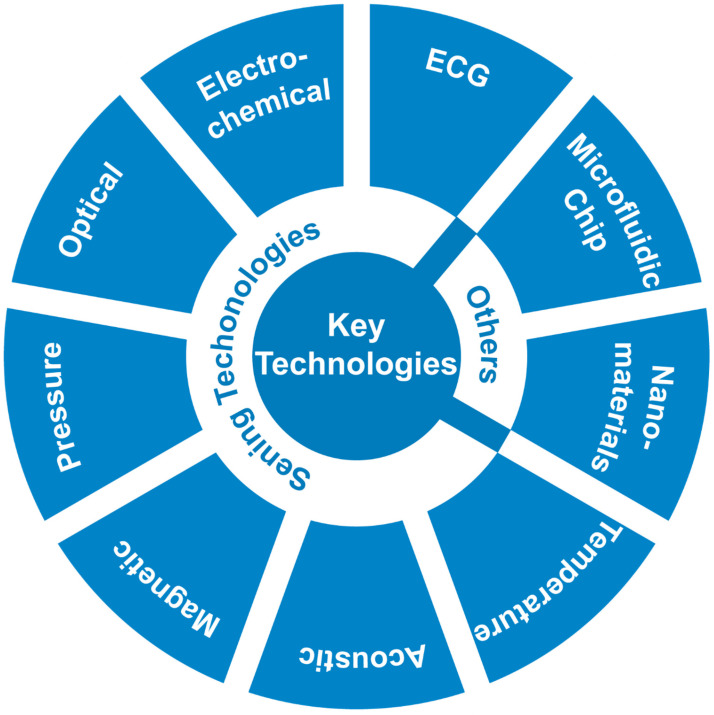
Key technologies that are used for dynamic monitoring.

Based on the sensor principle classification [40], this article will further explore its dynamic monitoring application in personalized drug therapy. Some studies of core sensing technologies such as electrochemical sensors [41], optical sensors [42], magnetic sensors [43], and pressure sensors [44], with their high sensitivity [45], rapid response [45], and non-invasive characteristics [46], not only enable real-time monitoring of drug concentrations [47], metabolites [48], and key indicators in the blood [28] but also assess the regulatory effects of drugs on cardiovascular function [49]. With the advancement of material science and microscale analytical techniques, these methods provide more precise and efficient solutions for drug efficacy evaluation and adverse reaction monitoring [50,51]. The application of these technologies (Figure 2) significantly enhances the precision and safety of cardiovascular drug treatments, offering patients more efficient and convenient monitoring methods [35,52].

### 2.1. Sensing Technologies

#### 2.1.1. ECG Sensor

Electrocardiogram (ECG) sensors are typically based on the principle of electrode detection, measuring a patient’s cardiac activity data (such as rhythm and waveforms) by recording the electrical signals generated by the heart with each beat [53]. They are often implemented as electrocardiographs, capable of collecting and analyzing ECG data in real-time. Clinically, ECG sensors are widely used to diagnose arrhythmias and other cardiac conditions, as well as to monitor the patient’s heart health [52]. Additionally, in the evaluation of cardiovascular drugs, ECG sensors can detect the effects of medications on cardiac electrical activity, helping to identify potential arrhythmias or cardiotoxic reactions [54].

Compared to indirect measurement sensors (such as devices that monitor peripheral physiological signals through photoplethysmography (PPG) sensors to indirectly measure cardiac activity), ECG sensors can directly detect the electrical activity of the heart, offering higher signal accuracy [55]. Continuous ECG monitoring of patients can more comprehensively capture individual variations in cardiac electrical activity. Currently, the commonly used clinical method, Ambulatory ECG Monitoring, enables continuous recording and analysis of patient data for 24 h or longer through portable devices (such as Holter monitors or wearable ECG sensors), providing data support for personalized treatment. This includes diagnosing arrhythmias, evaluating drug efficacy, or predicting cardiovascular events [56]. However, during the data collection process, signal quality may be affected by factors such as motion artifacts [57], changes in skin resistance [58], and environmental electromagnetic interference [59], which can disrupt the collection and analysis of personalized physiological data. For instance, signal artifacts caused by poorly adhered electrodes may be mistakenly identified by computer algorithms as atrial fibrillation (AF), potentially leading to misdiagnosis [60]. Therefore, optimizing sensor design and improving signal processing algorithms are effective approaches to enhancing the reliability and accuracy of the data.

ECG sensors, as an essential medical device, are widely used in hospitals and emergency settings with the traditional 12-lead ECG still serving as the gold standard for diagnosing cardiovascular diseases such as arrhythmia and myocardial ischemia. According to a relevant clinical guideline [61], the traditional 12-lead ECG continues to play a vital role in diagnosing atrial fibrillation (AF). At the same time, the guideline mentions the potential use of continuous monitoring devices, such as ambulatory ECG, for evaluating and diagnosing AF in selected patients, reflecting the personalized nature of AF diagnostic assessments. Furthermore, implantable ECG devices, such as implantable loop recorders, are mentioned as advanced technologies that may assist in detecting subclinical AF. For atrial tachycardia after pulmonary vein isolation, recent studies employing continuous implantable recorders to monitor surgical procedures have identified the incidence of early postoperative arrhythmia recurrence and confirmed a correlation between early and late recurrences [61]. This demonstrates the potential of continuous implantable recorders to help physicians gain a more comprehensive understanding of postoperative arrhythmia recurrence patterns, thereby providing a basis for more precise treatment and management. In addition, a clinical guideline [62] emphasizes the importance of selecting appropriate monitoring tools based on the specific conditions of different patients. For instance, for patients who have experienced systemic thromboembolic events but have no known history of atrial fibrillation, implantable cardiac monitors are considered a reasonable choice to improve detection sensitivity (Recommendation Class: 2a, Evidence Level: B-R). For patients requiring AF monitoring, the use of high-quality consumer ECG monitoring devices is recommended to track recurrence (Recommendation Class: 2a, Evidence Level: B-R) [62].

#### 2.1.2. Electrochemical Sensors

Electrochemical sensors achieve precise measurement of analytes by detecting changes in electrochemical signals, such as current, caused by target molecules [63]. In recent years, this technology has been widely applied in cardiovascular drug monitoring, particularly excelling in real-time monitoring of drug metabolism and key blood parameters [64]. Electrochemical sensors can detect the concentration of cardiovascular drugs (e.g., anticoagulants), providing critical guidance for optimizing drug dosages [65]. Additionally, they can monitor metabolites produced during drug metabolism, aiding in the assessment of patient responses to medications [66]. By immobilizing specific enzymes (e.g., glucose oxidase) on the sensor surface [67], electrochemical sensors can accurately detect metabolites produced during the metabolism of anticoagulants like warfarin [68]. In evaluating the effects of drugs on cardiac function, electrochemical sensors can monitor dynamic changes in electrolytes such as potassium and sodium ions in the blood in real-time [69,70]. Moreover, by detecting reactive oxygen species levels in the blood, these sensors can assess the regulatory effects of cardiovascular drugs on oxidative stress [71]. Notably, integrating electrochemical sensors into portable devices enables point-of-care testing (POCT), providing efficient and reliable technical support for clinicians to quickly adjust drug dosages [72].

Through an efficient electrochemical transduction mechanism, high-sensitivity and fast-response electrochemical sensors with clinical application potential can be developed. Romele et al. developed an ion sensing amplifier based on complementary organic electrochemical transistors (OECT), capable of real-time, multi-scale, high-sensitivity detection of ion concentration changes, with a sensitivity larger than 2300 mV V^−1^ dec^−1^ and a response time of 11 s [73]. Song et al. developed a wireless, non-invasive smart contact lens that integrates an electrochemical cholesterol biosensor with NFC wireless communication technology to enable real-time monitoring by detecting cholesterol levels in tear fluid. Animal experiments validated the correlation between cholesterol levels in tear fluid and those in blood, providing a novel non-invasive solution for the diagnosis and monitoring of hyperlipidemia and related cardiovascular diseases. Compared to traditional cholesterol detection methods, this smart contact lens offers a more convenient and continuous data support system for personalized monitoring through non-invasive, real-time cholesterol level tracking [74]. Electrochemical biosensing platforms play a critical role in precision medicine by utilizing biological receptors such as antibodies, aptamers, and enzymes to detect molecular biomarkers at different omics levels beyond metabolic markers. This enhances the resolution of individualized diagnosis and treatment for patients. With their design diversity and flexibility, these platforms can simultaneously detect drugs and biomarkers related to efficacy or toxicity (e.g., infection, inflammation, kidney function), thereby optimizing treatment plans and enabling personalized precision therapy [75]. Currently, electrochemical sensors in clinical cardiovascular disease measurements may be affected by the complexity of biological samples (e.g., interfering substances in the blood [76]) and sensor stability (e.g., sensitivity degradation due to prolonged use [76,77]), thereby affecting treatment. For instance, a decline in sensor stability (such as reduced sensitivity over time [76]) may lead to inaccurate follow-up data for patients, which, in turn, could affect physicians’ assessment of disease progression and adjustments to personalized treatment plans. Therefore, it is necessary to enhance measurement accuracy and reliability by optimizing sensor surfaces (e.g., anti-interference [78]) and improving sensor materials (e.g., using nanomaterials to enhance stability and sensitivity [77]).

Cardiovascular disease patients often have comorbid diabetes, making blood glucose monitoring critically important. Electrochemical sensors also play a significant role in this area, such as using electrochemical sensors to detect HbA1c [79]. According to a relevant international clinical guideline, diabetes screening is recommended for all cardiovascular disease patients, as undiagnosed diabetes is common in this population. The guideline points out the importance of optimizing blood glucose control after cardiovascular events and mentions the need for further research to determine whether optimizing blood glucose control in cardiovascular disease patients with diabetes, through technologies like continuous glucose monitoring, can improve clinical outcomes. Implementing various blood glucose management strategies is particularly important for diabetic patients at high cardiovascular risk. These strategies include setting personalized HbA1c targets, minimizing hypoglycemic episodes, and limiting blood glucose fluctuations [80]. This underscores the significant potential of developing electrochemical sensors for continuous HbA1c monitoring in advancing personalized blood glucose control in the future.

#### 2.1.3. Optical Sensors

Optical sensors analyze target molecules by detecting changes in light absorption, reflection, scattering, or fluorescence signals [81]. In recent years, these sensors have been widely applied in evaluating the efficacy of cardiovascular drugs, demonstrating the significant value of their potential application [82]. They can assess the regulatory effects of lipid-lowering drugs on cholesterol and lipid levels in the blood [83], while fluorescence labeling techniques enable dynamic tracking of drug distribution and metabolism within the body [84]. For example, fluorescence labeling can be used to monitor the improvement of vascular endothelial function caused by antihypertensive drugs [85]. Additionally, integrated into wearable devices, optical sensors can also provide real-time monitoring of heart rate and blood oxygen levels, indirectly assessing the cardiovascular protective effects of drugs [86].

Compared to electrochemical sensors [87], optical sensors do not require the use of chemical reagents or electrolyte solutions, avoiding the complexity of chemical reactions while enabling non-invasive operation [88]. Detection of cTnI can be used to identify late-stage AMI patients. Yu Li et al. developed an ultrasensitive detection method based on aptamer-based rolling circle amplification (RCA) and graphene oxide-based fluorescence resonance energy transfer (GO-FRET). This method can detect cardiac troponin I (cTnI) with a detection limit of 14.40 pg/mL, offering high sensitivity and specificity, making it suitable for clinical sample analysis [89]. In addition, it is reported that a graphene quantum dots fluorescence resonance energy transfer (FRET)-based sensor was developed for the rapid detection of the myocardial infarction (heart attack) biomarker cTnI. This sensor achieves specific detection through the covalent conjugation of antibodies with GQDs, with a detection limit as low as 0.192 pg/mL and a detection time of only 10 min, featuring high sensitivity and rapid response [90]. The high sensitivity and rapid response of this technology are of great significance for achieving early and accurate diagnosis of myocardial infarction, as well as providing timely and targeted treatment plans for patients. Overall, factors affecting the measurement accuracy of optical sensors mainly include light interference from the environment and potential interfering substances in the sample (such as non-target molecules or impurities) [91]. Effective solutions to these interference factors (such as light shielding techniques [92] and enhanced specificity recognition [93]) can significantly improve the accuracy and reliability of measurement data, providing individuals with more precise information. Among these, optical fiber sensors, by integrating recognition elements, can efficiently detect disease-related biomarkers, enabling real-time diagnosis. The flexibility and compactness of optical fibers allow them to support wearable or implantable devices, enabling continuous monitoring of human health information and showing promising prospects in precision medicine. However, fiber-optic biosensors face challenges such as low throughput, bulky device size, and potential risks of harm to the human body. In the future, driven by advancements in medical learning algorithms, novel materials, and optical engineering, next-generation fiber-optic sensors are expected to deliver superior diagnostic and therapeutic performance, better meeting the demands of precision medicine [94].

Myocardial infarction (heart attack) is commonly associated with coronary artery diseases, including acute coronary syndrome (ACS) [95]. Optical sensors can be used to detect related biomarkers (such as cTnI). A relevant clinical guideline [96] mentions the importance of high-sensitivity cardiac troponin (hs-cTn) detection in assessing patients with ACS. Additionally, the guideline mentions there is a trial [97] utilizing intravascular ultrasound (IVUS) and optical coherence tomography (OCT) to guide percutaneous coronary intervention (PCI), which, compared to angiography-guided approaches, can reduce target vessel failure. The study and practice demonstrate that optical sensors not only play a critical role in detecting biomarkers (such as cTnI) in ACS patients but also show extensive application and development prospects in intravascular imaging and interventional treatment guidance. This multidimensional application further underscores the potential of optical technology in the diagnosis and treatment of cardiovascular diseases [96].

#### 2.1.4. Pressure Sensors

Pressure sensors are typically based on principles such as piezoelectricity [98], capacitance [99], or strain gauges [100] and are used to monitor pressure changes within blood vessels or heart chambers, thereby evaluating the therapeutic effects of cardiovascular drugs [101]. Commonly used clinical pressure sensors include catheter-based pressure sensors [102] and cuff-based blood pressure sensors [103]. In addition, implantable pressure sensors [104] have also demonstrated certain application potential. For instance, implantable pressure sensors enable long-term monitoring of intracardiac pressure, thereby having the potential to evaluate the efficacy of drugs for heart failure [104].

Compared to optical and magnetic sensors, pressure sensors are characterized by their direct contact with physical pressure, making them suitable for contact-based measurements and capable of accurately reflecting changes in gas or liquid pressure [105]. Li et al. developed a high-performance flexible pressure sensor based on the soft strain effect, utilizing a flexible conductive film stuck on a hollow electrode as a soft strain gauge to generate and amplify tensile strain. This sensor can be used to detect pulse wave signals from deep and peripheral arteries. It features ultra-high sensitivity (~636.8 kPa^−1^), a wide detection range (0.1–56 kPa), and excellent stability (no significant performance degradation after 10,000 cycles at 10 kPa). It addresses the issue of sensitivity reduction under high pressure in traditional pressure sensors, providing a new approach for the early diagnosis of cardiovascular diseases and health monitoring [106]. The hemodynamic signals of peripheral arteries tend to weaken as blood flows toward the extremities, primarily due to the progressive narrowing of blood vessels and the rise in flow resistance. Moreover, for patients with peripheral vascular diseases, their blood flow characteristics are often more complex, which places higher demands on the sensitivity of sensors [107]. Sensors with excellent performance (ultra-high sensitivity and wide detection range [106]) are expected to address the limitations of traditional devices in detecting weak signals and the inconvenience of long-term monitoring. These sensors offer new possibilities for real-time, high-precision monitoring of pulse waves and blood pressure, enabling more sensitive capture of peripheral arterial signals. This contributes to the early diagnosis of cardiovascular diseases such as atherosclerosis, helps avoid missed diagnoses caused by insufficient detection, and provides reliable support for personalized health management. In addition, the existence of white-coat hypertension and masked hypertension further highlights the importance of sensors capable of precise long-term blood pressure monitoring. These sensors can provide technological support for better addressing individual differences in hypertension diagnosis [108]. Currently, pressure sensor measurements are influenced by factors such as the balance between sensitivity and detection range [109], mechanical performance and stability [110], as well as material and structural design [111]. In the future, through the optimization of materials [112], structural design [113,114], and signal processing technologies [115], it is expected that the measurement accuracy and stability of pressure sensors can be significantly improved, meeting the needs of multi-scenario applications.

A relevant clinical guideline [116] mentions that wearing an ambulatory blood pressure monitor (ABPM) is commonly used to supplement blood pressure readings obtained in a clinical setting. The device is typically configured to measure blood pressure every 15 to 30 min during the day and every 15 min to 1 h at night [117]. ABPM provides estimates of average blood pressure over the monitoring period, as well as daytime and nighttime averages. It can also identify the extent of nocturnal ‘dipping’, identify the early-morning BP surge pattern, estimate BP variability, and help identify symptomatic hypotension [118]. Moreover, many newer oscillometric devices can automatically inflate multiple times (at intervals of from 1 to 2 min), reducing disruption to the patient. While traditional methods remain dominant, there is growing evidence supporting the use of automated office blood pressure measurement techniques [119]. Additionally, the guideline recommends the use of ABPM or HBPM (home blood pressure monitoring) in specific cases. For example, for adults with white coat hypertension, regular monitoring using ABPM or HBPM is recommended to detect progression to sustained hypertension (Class of Recommendation [COR]: IIa, Level of Evidence [LOE]: C-LD) [116]. This reflects a shift in blood pressure monitoring from traditional office-based measurements to dynamic and automated methods, with greater emphasis on improving continuity and accuracy in measurements. Furthermore, by utilizing dynamic blood pressure monitoring and automated devices, it becomes possible to more precisely capture individual blood pressure variation patterns, thereby guiding personalized treatment plans.

#### 2.1.5. Magnetic Sensors

Magnetic sensors, by detecting magnetic nanoparticles or changes in magnetic fields, have become a cutting-edge technology for the precise identification of target substances, molecules, or biomarkers [120]. Clinical magnetocardiography (MCG) [121] and magnetoencephalography (MEG) [122]) can be used to detect weak biomagnetic fields. These techniques evaluate the activity of the heart and brain by detecting the subtle magnetic fields generated by their electrical activity, with magnetic sensors being an indispensable component of these technologies [123].

Previously, superconducting quantum interference devices (SQUIDs) have been a key technology supporting MCG. Magnetoresistive (MR) sensor technology has now achieved sensitivities suitable for MCG. Magnetoresistive materials exhibit resistance changes in response to external magnetic fields, and, when incorporated into sensor designs, they can provide extremely high sensitivity, reaching 5146%/mT [124]. In addition, it is reported that a high-sensitive 1D photonic crystal magnetic field sensor leverages Fano/Tamm resonance in the far-infrared region, offering new possibilities for magnetic field monitoring in the biomedical field. This sensor demonstrated a maximum sensitivity of 57 nm/Tesla under magnetic field strengths ranging from 20 to 140 Tesla [125]. Such high sensitivity reflects the sensor’s strong responsiveness to changes in magnetic field strength, showcasing its great potential in detecting subtle magnetic field variations. High-sensitivity magnetic sensors hold promising prospects in personalized medical devices, such as detecting weak biomagnetic fields, enabling more precise diagnostic and therapeutic solutions. However, due to their high sensitivity to magnetic field changes, magnetic sensors are prone to electromagnetic interference (EMI). To mitigate the impact of external EMI, shielding designs can be employed [126].

Magnetic sensors also serve as critical components in magnetic resonance imaging (MRI) systems, assisting MRI equipment in achieving high-precision imaging [127]. MRI is frequently mentioned in clinical guidelines [128] as an important tool for diagnosing and managing cardiomyopathies. The guideline further mentions that newly developed rapid cardiac magnetic resonance (CMR) techniques allow even very young children to undergo scans without the need for general anesthesia [128,129]. This indicates that, with advancements in rapid CMR technology, MRI will further enable non-invasive and safe diagnostics for special populations, such as children, expanding its applications in personalized medicine. At the same time, the related component—magnetic sensors—has the potential to evolve accordingly.

#### 2.1.6. Acoustic Sensors

Acoustic sensors can evaluate the efficacy of cardiovascular drugs indirectly by detecting changes in the propagation characteristics of sound waves (e.g., velocity, frequency, or echoes) within blood vessels or the heart, reflecting hemodynamic parameters or tissue conditions [130]. In recent years, acoustic sensors have been increasingly applied in the diagnosis and treatment of cardiovascular diseases, owing to their non-invasive nature, high sensitivity, and real-time monitoring capabilities, showcasing tremendous potential for further development [131].

Compared to traditional magnetic field sensors, acoustic sensors are less susceptible to interference in strong magnetic fields or complex electromagnetic environments, such as near MRI equipment, thereby maintaining high sensitivity [132]. A highly sensitive acoustic sensor based on electrospun piezoelectric nanofibers that utilizes the principle of converting sound waves into electrical signals has been reported. This sensor can detect low-frequency sound waves and noise with a sensitivity of up to 266 mV Pa^−1^, which is five times higher than that of traditional piezoelectric PVDF films [133]. A developed wearable ultrasound device was reported that employs a piezoelectric transducer array and a deep learning model to enable real-time, continuous cardiac function assessment. It can measure key cardiac performance indicators, such as left ventricular ejection fraction and cardiac output, making it suitable for dynamic cardiovascular monitoring and stress testing. The device demonstrates high consistency with commercial equipment in measuring critical cardiac indicators (e.g., ejection fraction and cardiac output) with minimal discrepancies. Its dynamic range reaches 63.2 dB, exceeding the 60 dB threshold required for medical diagnostics, indicating excellent resolution of contrast between different tissues [134]. The high dynamic range capability allows for clear differentiation of subtle contrasts between cardiac tissue and low-reflectivity structures such as blood [135], ensuring precise detection of pathological regions and functional abnormalities and providing richer imaging details in personalized cardiovascular disease diagnosis and treatment [136]. This portable, high-precision sensor, capable of continuous and real-time cardiac function monitoring, not only exhibits strong applicability in daily health monitoring but also provides high-quality and reliable individual data for personalized cardiovascular disease management. Overall, factors currently affecting acoustic sensor measurements include the sensor’s placement [137], environmental noise levels [138], patient activity status [139], and calibration accuracy [140]. Optimizing sensor design and performance to address these factors has become a key challenge in enhancing their effectiveness in personalized monitoring. Among them, wearable acoustic sensors, due to their portability, are advantageous for long-term monitoring. Future challenges include improving material biocompatibility and performance, further miniaturizing sensor designs, and establishing human sensor networks [141].

Echocardiography is a core tool for assessing cardiac structure and function, aiding in the confirmation of heart failure diagnoses [142]. In a clinical guideline for the diagnosis and treatment of acute and chronic heart failure, transthoracic echocardiography is recommended as a key diagnostic test for evaluating cardiac function in all patients with suspected chronic heart failure (Class I recommendation, Level of Evidence C) [143]. Additionally, other relevant guidelines point out that for patients presenting cardiogenic shock, hemodynamic instability, or suspected mechanical complications, urgent ultrasound examinations should be performed, potentially including bedside ultrasound assessments conducted by properly trained clinicians [96,144]. This reflects the critical role of bedside ultrasound in rapid diagnosis and evaluation. With its real-time capability and portability, bedside ultrasound is expected to play an even greater role in personalized medicine in the future.

#### 2.1.7. Temperature Sensors

Temperature sensors detect temperature changes or thermal conductivity properties to indirectly reflect physiological parameters related to cardiovascular drug effects, such as blood flow, tissue metabolism, or inflammatory responses, thereby enabling efficacy monitoring [145]. In recent years, these sensors have gained increasing attention in the research and application of cardiovascular disease treatment [146].

The working principle of temperature sensors differs from that of sensors that rely on receiving reflected signals [147,148]. Temperature sensors typically measure temperature by directly contacting the object or environment being measured to sense changes in thermal energy. However, some non-contact temperature sensors achieve measurement by detecting infrared radiation [149]. With technological advancements, the application of flexible temperature sensors has become increasingly widespread [147]. Deng et al. developed a flexible wearable thermal sensor based on the principle of thermal anemometric flow, which can be used for continuous monitoring of blood flow changes in the vascular access of hemodialysis patients. This sensor detects changes in blood flow through a non-invasive method, featuring rapid response capabilities (with an overall response time ranging from a few seconds to several tens of seconds), and has demonstrated its practicality in both human and animal experiments [150]. In comparison, one of the commonly used clinical techniques, the thermodilution method used in right heart catheterization (RHC), is one of the important techniques for measuring cardiac output (CO) and is based on the principle of temperature sensing. It typically involves invasive procedures [151]. Compared to traditional techniques, the non-invasive sensor with rapid response and continuous monitoring holds significant application value in the personalized diagnosis and treatment of cardiovascular diseases. Its non-invasive design helps improve patient compliance, and its monitoring precision and timeliness provide support for early intervention, thereby enhancing the patient treatment experience and optimizing outcomes [152,153]. Key factors currently affecting temperature sensor monitoring include the thickness and thermal conductivity of the vessel wall, the thickness of the skin layer, and variations in blood flow rate [154,155]. In addition, for flexible temperature sensors, geometric design and attachment position significantly influence monitoring effectiveness [156,157,158].

In a clinical guideline [143] for the diagnosis and treatment of acute and chronic heart failure, RHC is recommended for patients with heart failure thought to result from constrictive pericarditis, restrictive cardiomyopathy, congenital heart disease, or high output states (class IIa). Additionally, it can be considered for selected patients with HFpEF to aid in confirming their diagnosis (class IIb) [143]. This reflects the importance of RHC in the diagnosis of heart failure, as well as the critical role of related sensor technologies (such as temperature sensors), which directly impact the accuracy and reliability of diagnostics.

### 2.2. Microfluidic Chip Technology

Microfluidic chip technology manipulates fluid flow on a microscale chip to precisely simulate biochemical reactions and metabolic processes within the body [159]. In recent years, it has been widely applied in cardiovascular drug screening [160] and metabolism research [161], playing an especially important role in personalized medicine.

Microfluidic chips significantly enhance the efficiency of drug screening and monitoring while reducing reliance on animal experiments, providing a powerful tool for new drug development [162]. For example, by simulating liver metabolism on the chip [163,164,165], researchers can study the effects of antihypertensive drug metabolites on blood vessels. Through the design of microchannels on the chip, the technology can detect the impact of drugs on the adhesion [166] and migration capabilities [167] of vascular endothelial cells. Additionally, with multi-channel microfluidic systems, it is possible to investigate interactions between drugs and various proteins, enabling a comprehensive evaluation of drug safety [168,169]. This technology not only advances drug development but also lays a solid foundation for precision medicine and personalized treatment [170,171]. Microfluidic technology (LOC devices) combined with induced pluripotent stem cells (iPSCs) can generate personalized cardiovascular models. This integration enables researchers to simulate real cardiovascular environments on a chip, facilitating the study of disease mechanisms and drug screening. Through this technology, drug screening and efficacy prediction can be tailored to the specific biological characteristics of individual patients, thereby optimizing treatment plans. It provides a powerful tool and platform for achieving precision medicine and personalized cardiovascular drug therapy [172].

### 2.3. Nanomaterials Technology

Nanomaterials not only demonstrate great potential in the field of novel cardiovascular therapeutic drugs but also serve as a powerful tool for enhancing sensor performance [173]. The unique physicochemical properties of nanomaterials, such as nanoparticles, nanotubes, or quantum dots, significantly enhance the performance of sensors [174]. In recent years, nanomaterials have been widely applied in the development of cardiovascular drug sensors [175,176], greatly improving their sensitivity and selectivity [176].

The integration of nanomaterials allows sensors to detect target molecules at ultra-low concentrations, advancing the early diagnosis of cardiovascular diseases and the development of personalized treatments [177,178,179]. For instance, these materials enable the detection of cardiovascular drugs, such as statins, at extremely low concentrations, thereby optimizing drug dosage [180]. Incorporating gold nanoparticles into electrochemical sensors enhances the sensitivity for detecting anticoagulant drugs [181]. Sensors modified with carbon nanotubes can monitor real-time changes in blood cholesterol levels with enhanced sensitivity [182].

## 3. Dynamic Monitoring Based on Individual Differences

**Figure 3:**
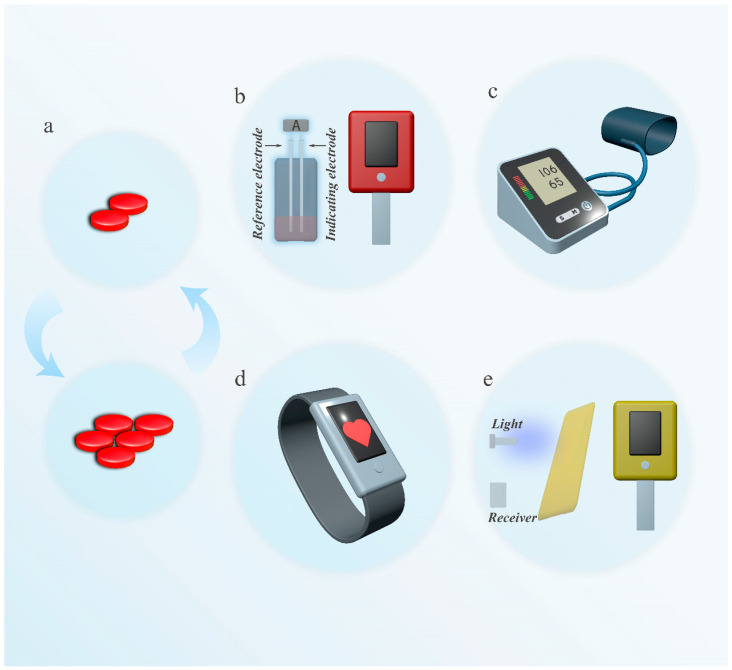
Examples of common dynamic monitoring to adjust medication for cardiovascular disease. (**a**). Schematic diagram of optimizing dosage. (**b**). Measurement of INR using a coagulation analyzer (left image: illustrating possible electrochemical principles) (**c**). A blood pressure monitor used to measure blood pressure. (**d**). An electronic watch that monitors heart rate. (**e**). Cholesterol meter for measuring cholesterol.

Cardiovascular drugs are mainly categorized into anticoagulants [183], antihypertensives [184], antiarrhythmics [185], and lipid-lowering agents [186], and other classes, which achieve therapeutic effects by regulating blood coagulation [187], blood pressure control [32], cardiac electrical activity [188], and lipid metabolism [189]. These drugs are suitable for patients with thrombotic diseases [190], hypertension [191], arrhythmias [192], and hypercholesterolemia [193], helping to reduce the risk of cardiovascular events. When using these medications, due to individual differences (such as genetics, metabolic capacity, and disease conditions), it is essential to consider the effective dosage and potential adverse reactions. Therefore, during treatment, measuring biomarkers or physiological parameters related to efficacy and adverse reactions in the patient’s body is crucial (Figure 3) [194,195,196].

### 3.1. Dynamic Monitoring of Anticoagulant Drugs

#### 3.1.1. Mechanism of Action and Metabolic Pathways of Warfarin

Warfarin is a classic oral anticoagulant whose mechanism of action involves inhibiting vitamin K epoxide reductase-VKORC1, thereby disrupting the synthesis of coagulation factors II, VII, IX, and X to achieve an anticoagulant effect [197,198]. It is widely used for the prevention and treatment of various thrombotic disorders, including deep vein thrombosis [199], pulmonary embolism (PE) [200], and thromboembolism prevention [201] in patients with atrial fibrillation, and thrombosis risk management following artificial heart valve replacement surgery [202].

However, warfarin’s metabolic pathway is complex and primarily depends on the cytochrome P450 enzyme (CYP2C9) in the liver [203]. The efficiency of CYP2C9 metabolism is significantly influenced by genetic polymorphisms, leading to considerable individual variability in metabolic capacity based on genotype [204,205]. This variability results in substantial differences in warfarin concentrations in the bloodstream, making its therapeutic window extremely narrow [16,206]. Dose adjustments must be made with great caution: excessive dosing can lead to severe bleeding risks, while underdosing may fail to effectively prevent thrombus formation [207,208]. Thus, monitoring warfarin’s pharmacodynamics is critical to ensuring both safety and efficacy [209].

#### 3.1.2. Dynamic Monitoring of INR Values

The International Normalized Ratio (INR) is a crucial indicator for evaluating the anticoagulant effect of warfarin [210], and its monitoring is essential for optimizing treatment outcomes and reducing the risk of bleeding or thrombosis [211]. Electrochemical sensors (Figure 3b) achieve real-time monitoring of INR values by detecting the activity of coagulation factors in the blood. These sensors rely on electrical signals generated from reactions on the electrode surface, enabling rapid and accurate reflection of coagulation status. Portable electrochemical sensor devices have been widely used in clinical and home monitoring, assisting patients and doctors in timely adjustments of warfarin dosages to reduce the risks of bleeding or thrombosis [212]. Williams et al. developed a fully printed portable prothrombin time (PT) sensor based on the principle of electrochemical impedance detection. This low-cost, rapid, and precise point-of-care testing (POCT) system is designed for monitoring blood coagulation status. By optimizing the frequency and using flexible substrates, the sensor can stably detect the blood coagulation process and shows potential for integration into wearable devices [213].

Optical sensors, on the other hand, monitor INR values, detecting changes in the optical properties of blood samples, such as absorbance, scattering [214,215,216]. In practical applications, optical detection techniques have been integrated into portable or bedside testing devices (e.g., optical coagulation analyzers) for routine patient monitoring [217].

Chan et al. developed a low-cost PT/INR detection system based on micro-mechanical motion tracking using a smartphone’s vibration motor and camera. This system employs the vibration motor to drive small copper particles in blood or plasma and uses the camera to capture changes in particle motion. By combining video analysis algorithms, the system detects changes in sample viscosity during coagulation, enabling measurements of coagulation time and INR values. This innovative approach provides an economical and practical testing solution for resource-limited settings. This is achieved by enabling the system to operate on older smartphones, such as second-hand iPhone 5s devices released in 2013, which are priced at just $35. By making coagulation testing more accessible, this method has the potential to improve the time within the therapeutic range for anticoagulation users, especially in rural areas [218].

### 3.2. Dynamic Monitoring of Antihypertensive Drugs

#### 3.2.1. Mechanism of Action of β-Blockers

β-blockers are a class of drugs that exert their effects by blocking β-adrenergic receptors [219]. Their primary mechanism involves reducing heart rate and myocardial contractility, thereby decreasing myocardial oxygen consumption and effectively controlling blood pressure [220]. These drugs are widely used in the treatment of hypertension [221], angina pectoris [222], and heart failure [223], significantly improving patient outcomes [224].

However, the therapeutic effects of β-blockers vary among individuals due to differences in patient characteristics, and dose adjustments must be made with particular caution. Excessive doses may lead to hypotension [225] or bradycardia [226], while insufficient doses may fail to achieve the desired therapeutic effect. Therefore, monitoring physiological indicators such as heart rate and blood pressure is crucial for optimizing treatment regimens, enhancing efficacy, and reducing the risk of adverse effects [227].

#### 3.2.2. Dynamic Monitoring Heart Rate and Blood Pressure Data

Indicators (including uncommon indicators) for evaluating the effects of β-blockers include heart rate [228], blood pressure [229], heart rate variability [230], cardiac output [231,232], lactate levels [233], and ECG [234] parameters. Monitoring techniques can assess the drug’s effectiveness to a certain extent by focusing on core indicators. By tracking changes in patients’ heart rate and blood pressure (Figure 3c), scientific evidence can be provided for adjusting β-blocker dosages [235]. For instance, portable blood pressure monitoring devices [236] and wearable heart rate sensors [86] can record patients’ physiological data and medication dosages can be optimized through algorithmic analysis [237]. Mai et al. developed an intelligent drug infusion system by combining active disturbance rejection control and deep reinforcement learning in a closed-loop control method, achieving precise regulation and automated management of postoperative patients’ mean arterial pressure [238].

### 3.3. Dynamic Monitoring of Antiarrhythmic Drugs

#### 3.3.1. Mechanism of Action of Antiarrhythmic Drugs

Arrhythmias are often closely related to abnormalities in cardiac electrical activity, such as disruptions in the generation or conduction of action potentials in myocardial cells, leading to excessively fast, slow, or irregular heart rhythms [239,240]. The treatment of arrhythmias primarily relies on regulating the function of ion channels in myocardial cells. For example, sodium channel blockers can slow the rate of action potential upstroke [241,242], while calcium channel blockers can reduce the excitability and conductivity of myocardial cells [243,244], thereby restoring normal cardiac rhythm. However, the complexity and variability of arrhythmic conditions [245,246] may lead to imprecise control of the disease. Therefore, monitoring cardiac electrical activity is necessary to guide the use of medications.

#### 3.3.2. Dynamic Monitoring of Heart Rhythm

The treatment of arrhythmias and the optimization of drug dosages often rely on the monitoring of multiple cardiac electrophysiological parameters [247]. Generally, monitoring technologies assess the state of a patient’s cardiac electrical activity by recording key parameters such as heart rate (Figure 3d) [248], rhythm [249], ECG waveforms [250], and heart rate variability [251]. These indicators reflect the rhythmicity, conductivity, and stability of electrical activity in the heart, aiding physicians in analyzing the type, severity, and dynamic trends of arrhythmias [252]. This provides a scientific basis for adjusting the dosage of antiarrhythmic drugs [253].

The emergence of devices such as Holter monitors and wearable ECG sensors has made real-time, continuous heart rhythm data collection possible [254]. These devices not only capture the frequency and severity of arrhythmias but also provide crucial references for personalized medication management [255,256]. For instance, in patients with atrial fibrillation, heart rhythm data monitored by sensors can guide the dosage of antiarrhythmic drugs (e.g., amiodarone), helping to avoid risks such as bradycardia or loss of rhythm control [257,258,259].

### 3.4. Dynamic Monitoring the Efficacy of Lipid-Lowering Drugs

#### 3.4.1. Mechanism of Action and Adverse Reactions of Statins

Statins are used to lower low-density lipoprotein cholesterol (LDL-C) levels in the blood, with the aim of preventing and treating atherosclerotic cardiovascular disease (ASCVD) [260,261,262]. Their primary mechanism involves the inhibition of HMG-CoA reductase, which reduces cholesterol synthesis, thereby achieving lipid-lowering effects [263]. Although statins play a crucial role in both primary and secondary prevention of cardiovascular diseases [264,265], their use may lead to adverse reactions such as rhabdomyolysis [266,267,268]. These adverse effects are closely associated with elevated creatine kinase (CK) levels [269,270]. Therefore, during treatment, it is essential to regularly monitor patients’ cholesterol and CK levels to ensure both the efficacy and safety of the medication [270,271].

#### 3.4.2. Dynamic Monitoring of Cholesterol Level

The techniques used to monitor cholesterol levels include various types [272,273], among which optical sensors (Figure 3e) have demonstrated significant application value in cardiovascular disease management due to their high sensitivity and specificity [274]. Optical sensors can rapidly evaluate cholesterol levels by detecting the absorbance or fluorescence signals of cholesterol molecules in the blood [275,276,277]. This sensor technology can be integrated into portable detection devices, providing cholesterol data for patients and doctors, thereby aiding in the optimization of statin dosage adjustments [277].

Ni et al. synthesized a novel fluorescent compound, FDD (fluorescent digitonin derivative), which achieves non-invasive detection of skin cholesterol by specifically binding to cholesterol and emitting fluorescence signals. This method can be used for atherosclerosis risk assessment. By exciting cholesterol-bound FDD with 405 nm light and detecting fluorescence intensity changes, cholesterol levels can be quantitatively analyzed [278].

#### 3.4.3. Dynamic Monitoring of Creatine Kinase Levels

To promptly detect the potential risk of rhabdomyolysis associated with statin use in the treatment of atherosclerotic cardiovascular disease (ASCVD), monitoring technologies can be employed to track CK (creatine kinase) levels [279]. Among these, electrochemical sensors [280,281] and optical sensors [282,283] are ideal monitoring tools due to their high sensitivity and rapid response capabilities.

Electrochemical sensors have the advantage of enabling precise measurements of CK [279,284], while optical sensors offer non-invasive assessment [285]. These sensor technologies enable monitoring of CK levels, offering critical support for the early warning of rhabdomyolysis [284]. By dynamically tracking changes in CK levels, physicians can adjust medication dosages or switch treatment plans in time, thereby maximizing patient safety during drug administration.

## 4. Multi-Dimensional Data Integration and Analysis

The integration of multidimensional data holds significant importance in modern medicine, particularly in the diagnosis and treatment of cardiovascular diseases, where its value is increasingly evident [286]. By comprehensively integrating various layers of data—such as biomarkers, omics data, and external factors like environment and lifestyle—it is possible to reflect a patient’s health status holistically, from the molecular to the individual level [287]. In modern healthcare, with the growing demand for personalized medicine, data integration technologies are gradually becoming one of the key drivers for optimizing drug therapies and achieving precision medicine. Effectively integrating multidimensional biological data to support the optimization of drug therapies has become a critical focus of research [288,289].

**Figure 4:**
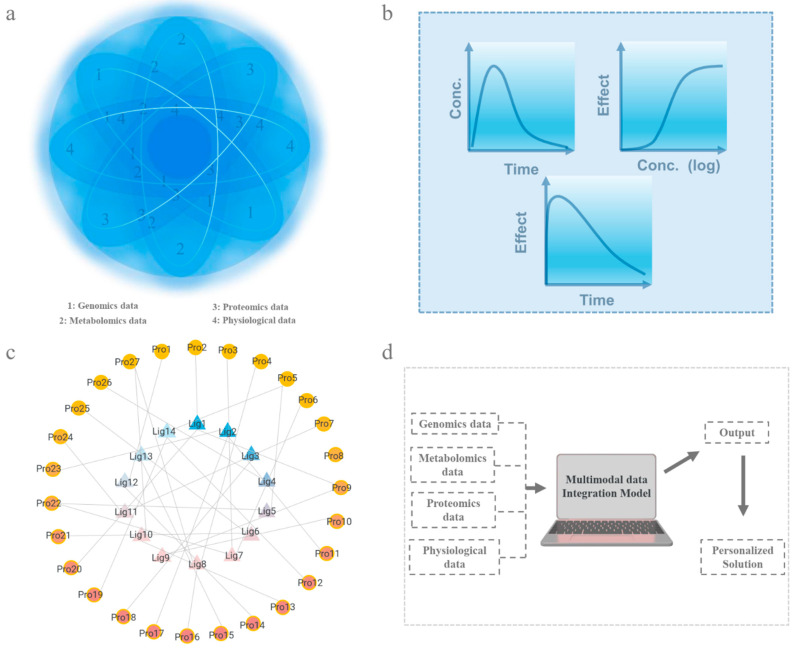
Integration and analysis of multidimensional data. (**a**). A schematic diagram of the integration and analysis of multidimensional data. (**b**). Schematic diagram of PK/PD model (Con.: Concentration). (**c**). Schematic diagram of drug–target network (Lig: ligand molecules, Pro: protein receptor). (**d**). Diagram of machine learning (multidimensional data integration and analysis through specific models).

In this context, pharmacokinetic and pharmacodynamic models (PK/PD models) serve as essential tools for data integration and have been widely used in drug dosage design and efficacy evaluation [290]. Meanwhile, drug–target networks provide a novel perspective for systematically analyzing the interactions between drugs and their targets. These network models not only optimize drug design and combination therapy strategies but also offer critical theoretical support for precision treatment [291]. Moreover, with the rapid advancement of artificial intelligence, machine learning has gradually become a pivotal tool in precision medicine. By leveraging multidimensional data, machine learning can uncover hidden correlations between datasets and dynamically predict drug responses and adverse effects, thereby providing scientific support for the development of personalized treatment plans (Figure 4a) [292]. The following sections will explore how PK/PD models, drug–target networks, and machine learning utilize multidimensional data integration to achieve more efficient precision medicine.

### 4.1. Pharmacokinetic/Pharmacodynamic Models

PK/PD models (Figure 4b) can be a core tool for optimizing drug therapy. PK models describe the processes of absorption, distribution, metabolism, and excretion of drugs in the body, while PD models reveal the mechanisms of drug action on their targets [293]. However, the accuracy of these models heavily relies on high-quality data, and individual variability (e.g., genetic profiles and metabolic characteristics) [294] may limit their applicability to all patients. Furthermore, in complex pathological conditions, the application of these models remains constrained, as they may not fully account for drug interactions in cases of multiple comorbidities [295,296].

However, analyzing PK and PD models only may overlook the limitations in achieving a comprehensive understanding of individual responses to drug actions. By further integrating multidimensional measurement data, these models have the potential to quantitatively describe the behavior and mechanisms of drugs within the body [297,298]. Such models can also be used to predict drug efficacy or adverse reactions [297]. The models thus established can not only help to understand the processes of drug action but also guide personalized medicine and optimize treatment regimens. Individual differences in drug metabolism rates due to genetic factors pose significant challenges to precision medicine [299]. With the development of genomics and metabolomics, integrating patients’ genetic information with PK/PD models offers a novel solution for personalized healthcare [300]. A patient’s genome can reveal gene variations related to drug metabolism and targets [301], while physiological data and biomarkers further reflect individual differences in metabolism rates, drug clearance, and target sensitivity [302]. Moreover, the application of sensor technology enables continuous monitoring of patients’ physiological parameters [303]. These high-frequency, accurate data not only capture the dynamic changes in a patient’s condition but also record immediate physiological responses, providing critical evidence for personalized treatment [303]. For example, in cardiovascular disease management, PK/PD models that combine genomic information with real-time monitoring data have the potential to significantly enhance drug efficacy and reduce the incidence of adverse reactions. The application of this technology not only provides a safer and more effective solution for the precise treatment of cardiovascular diseases but also lays the foundation for remote healthcare and intelligent management of chronic diseases, further advancing the development of personalized medicine [300].

### 4.2. Drug–Target Networks

Drug–target networks (Figure 4c) are a tool that constructs network models of drug–target relationships to systematically analyze drug mechanisms of action and target interactions [304]. They are based on the interactions between drugs and targets, using network topology to uncover multi-target mechanisms in complex diseases, thereby optimizing drug design and combination strategies [291,305]. The complex topology of drug–target networks reflects the polypharmacology of many drugs [306]. For example, statins (such as atorvastatin) act on the key target HMG-CoA reductase, effectively lowering cholesterol levels. Furthermore, it has been revealed that the potential anti-inflammatory and vascular function-improving effects of statins offer more precise guidance for clinical medication [307]. In cardiovascular drug development, drug–target networks may indicate potential disease targets, enabling more drug candidates [308]. Additionally, it can analyze the potential of multi-drug combinations to optimize combination therapy and enhance therapeutic outcomes. In clinical applications, drug–target networks can assist in evaluating the target coverage of drugs and potential drug interactions, providing a scientific basis for personalized treatment [307].

As an essential tool in systems biology, drug–target networks play a crucial role in cardiovascular drug therapy. By integrating multi-level biological data, drug–target networks provide a comprehensive analysis of the complex interactions between drugs and biological systems, offering a scientific basis for personalized treatment [309,310]. Through network analysis, drug–target networks can be utilized to design combination therapy strategies targeting multiple sites, thereby achieving more effective therapeutic outcomes [311]. For instance, in complex diseases such as hypertension combined with diabetes, network analysis can uncover the complex relationships of different drugs to act on targets (e.g., insulin receptors and angiotensin receptors) while comprehensively evaluating possible drug–drug interactions [312,313]. This approach offers a novel perspective for precision medicine, enabling the development of more targeted combination therapies that not only enhance treatment efficacy but also minimize side effects, ultimately improving overall patient outcomes [314]. Theoretically, by combining real-time monitoring of biomarkers and drug–target networks, we can dynamically adjust treatment plans, significantly enhancing the precision and safety of drug therapy [297]. Moreover, drug–target networks have the potential to assess individual differences in drug metabolism and response by incorporating patient-specific metabolic enzyme activity or genetic polymorphisms [315]. In clinical application, this approach has the potential to quantify a patient’s efficiency in metabolizing cardiovascular therapy drugs while incorporating the expression levels of targets to comprehensively evaluate the actual effects of the drug in the body. This integration significantly enhances the precision of personalized drug dosage design. As a core tool for cardiovascular disease drug development and treatment optimization, drug–target networks provide innovative technical support and research perspectives for precision medicine.

### 4.3. Machine Learning

Machine learning (Figure 4d) is a technology that uses algorithms and statistical models to identify patterns and make predictions from data. Its advantages lie in its ability to quickly process massive amounts of data and extract valuable information while also possessing self-optimization and continuous learning capabilities, thereby improving prediction accuracy over time [316,317]. In the clinical field, machine learning has developed rapidly and is widely applied in disease diagnosis, treatment optimization, and personalized medicine, significantly enhancing medical efficiency and precision [318,319]. Currently, machine learning is being increasingly applied in cardiovascular treatment, including heart disease risk prediction [320], electrocardiogram anomaly detection [15], and personalized drug dosage adjustment [321], providing significant support for the precise treatment of cardiovascular diseases. For example, a relevant study has conducted continuous dynamic heart rhythm monitoring for 30 days on patients with stroke risk factors but no known atrial fibrillation, analyzing their ECG data using artificial intelligence (AI) algorithms. The research indicates that the artificial intelligence (AI) algorithm-guided targeted screening approach is more efficient than traditional methods and can improve the effectiveness of atrial fibrillation screening [322]. In addition, adverse reactions to cardiovascular drugs can lead to severe consequences, such as fatal bleeding caused by anticoagulants [323] or acute hypotension and arrhythmia induced by certain antihypertensive drugs [324], posing life-threatening risks to patients. Traditional methods often rely on clinical observation and patient self-reporting to identify adverse reactions [325]. However, these methods have difficulty capturing early physiological changes or individual differences, which may lead to missed opportunities for timely intervention. Machine learning has demonstrated exceptional potential in predicting drug responses and adverse reactions to help patients avoid drug-related risks and improve the safety and effectiveness of treatments [326,327].

In recent years, the application of deep learning, a subset of machine learning, in cardiology has become increasingly widespread, with its importance reflected in its powerful predictive capabilities. For instance, deep neural networks (DNNs), as a sophisticated form of artificial neural networks, have emerged as a powerful tool in the field of cardiology, capable of automatically learning features and making predictions from raw data. These models, characterized by their multi-layer input and output structures, excel at handling unlabeled and unstructured data. By extracting key features from various high-dimensional data sources, DNNs have played a crucial role in advancing data-driven discoveries [328]. At present, artificial intelligence has made considerable progress in the interpretation of medical imaging, but its integration into broader healthcare fields remains significantly limited. However, clinical practice often requires the integration of multimodal data for analysis, making the development of AI models capable of multidimensional data integration and analysis quite urgent [328]. As a commonly used learning technique in deep learning, the Transformer model has demonstrated excellent performance and significant potential in integrating multimodal data [329]. A study reported a Transformer model based on the self-attention mechanism, which utilized patient features from the Cleveland Heart Disease dataset (e.g., age, blood pressure, cholesterol, etc.) to predict cardiovascular diseases. Unlike traditional recurrent neural network (RNN) methods, this model leveraged the advantages of position-level attention mechanisms and self-attention layers to learn the representation of the entire sequence, enabling the identification and evaluation of the relative importance of various sequence components, thereby enhancing predictive effectiveness. Its predictive accuracy reached as high as 96.51% and demonstrated strong interpretability, aiding doctors in understanding key features [330]. Integrating patients’ genomics, metabolomics, proteomics data, and real-time physiological data can be a crucial method to improve treatment outcomes in cardiovascular personalized medicine. For example, although atrial fibrillation (AF), long QT syndrome (LQTS), and Brugada syndrome (BrS) have different underlying causes, and current guidelines do not yet recommend genetic testing for familial atrial fibrillation; it is important to note that the role of genetic testing in the personalized management of these arrhythmias is becoming increasingly significant. This is because pathogenic or likely pathogenic variants in multiple disease-causing genes can contribute to the development of the final arrhythmia phenotype [331]. Therefore, by integrating multi-dimensional data, like patient genomics or/and metabolomics information [332], with real-time physiological data, machine learning models such as the Transformer, which excel in multidimensional data integration and analysis, have the potential to achieve highly accurate predictions. Through deep learning and feature extraction techniques, they can uncover potential correlations among different data types [333]. For instance, deep learning models can effectively determine a patient’s sensitivity to specific drugs, assisting doctors in formulating more precise treatment plans [334,335]. However, the integration and standardization of multimodal data remain a major challenge. Due to the significant differences in the sources and formats of data across different dimensions, the process of unifying and analyzing these data is extremely complex. In addition, improving the interpretability of deep learning models is also a critical issue. In the diagnosis of cardiovascular diseases or the evaluation of drug efficacy, understanding the basis of model decisions is essential, as it directly impacts the credibility and practical value of these models in clinical applications [336].

## 5. Challenges and Future Prospects

### 5.1. Complexity of Cardiovascular Pharmacotherapy

The treatment of cardiovascular diseases is highly complex, requiring not only long-term medication but also managing adverse reactions such as drug side effects and resistance [337,338]. Moreover, due to the interplay of multiple pathological mechanisms, cardiovascular diseases are often accompanied by other related conditions such as diabetes, further complicating treatment [339,340]. Consequently, combination therapy has become a critical treatment strategy. However, the metabolic pathways of different drugs may interact with each other, potentially affecting efficacy and safety. Individual differences in genetic background, age, sex, and lifestyle lead to variations in drug absorption, metabolism, and excretion, resulting in significant differences in metabolite levels or potential risks among patients [315,341]. Future advancements in implementing personalized therapy aim to optimize drug selection and dosage adjustments based on the specific conditions of each patient. This approach can reduce the risk of drug interactions, enhance therapeutic efficacy and safety, minimize side effects, and improve patient compliance and quality of life [342,343].

### 5.2. Advances in Monitoring Technologies for Cardiovascular Drug Efficacy

Given the complexity of cardiovascular disease treatment, timely assessment and adjustment of drug regimens is an effective solution, with monitoring technologies serving as a critical tool to achieve this goal [342]. However, many current sensors still suffer from limitations in detection sensitivity and specificity, such as low accuracy in detecting specific biomarkers or limited ability to distinguish target molecules. These shortcomings not only hinder precise adjustments to drug regimens but may also lead to misjudgments and erroneous results [344]. To overcome these deficiencies, future research may focus on the development of novel materials and the optimization of sensor design to enhance sensor performance [345]. Additionally, due to drug–drug interactions, there is an increasing demand for the simultaneous detection of multiple biomarkers. The capability for multi-parameter synchronous detection is likely to become a key trend in the development of sensor technology [346,347]. Facing the complex data generated by individualized responses, integrating advanced data processing methods (e.g., machine learning) to comprehensively analyze and predict monitoring results holds promise for achieving precise combination therapy and personalized treatment [292].

Furthermore, optimizing the efficacy of cardiovascular disease treatment may also involve the application of implantable devices. Since implantable devices are in direct contact with the body, they offer higher accuracy in certain aspects [348,349]. However, during long-term use, these devices are often affected by the surrounding physiological environment, such as encapsulation, material aging, performance degradation, and energy leakage. These post-implantation challenges highlight the urgent need for advancements in new material development, optimization of surface functionalization technologies, and improvements in self-powering technologies [350,351,352,353]. By addressing these critical issues, the safety and widespread adoption of implantable devices in long-term use is expected to improve significantly, providing more reliable solutions for cardiovascular disease treatment [354].

### 5.3. Challenges and Solutions in Clinical Translation

Although sensor technology, as a rapidly developing and highly focused area in monitoring methods, has demonstrated excellent performance in laboratory settings, its clinical application validation still faces numerous challenges [355]. Firstly, there are significant differences in experimental design and data processing methods for current sensor monitoring, which affect the comparability and consistency of data in cardiovascular drug monitoring technologies. Therefore, establishing an analytical model to evaluate such data would help improve the efficiency of technology comparison and enhance the scientific rigor of performance evaluation [356,357]. Secondly, some sensors are tested in overly specific subjects or application scenarios, limiting their applicability to diverse patient groups and varied application contexts. Thus, developing technologies compatible with diverse populations and scenarios will become a trend. By integrating advanced data processing methods, these technologies can further enable personalized and precise treatments [292,358,359]. Finally, many studies remain at the preclinical stage and have yet to progress into extensive clinical trials. The simplified physiological environments simulated in vitro often fail to fully reflect the complex physiological dynamics in vivo, leading to significant differences in sensor performance between real biological environments and laboratory conditions [360]. Therefore, further evaluation of their stability and reliability in complex bodily fluid environments is necessary. To promote the clinical translation of sensor technologies, combining standardized yet diversified experimental designs with validation in complex bodily fluid environments may become a key solution [292,357,361].

### 5.4. Development Direction of Cardiovascular Drug Therapy

To achieve personalized treatment, dynamically monitoring the impact of cardiovascular drugs on individuals’ multi-level physiological indicators may be a key solution, with data processing being an indispensable part of this process [362,363]. The challenges in these areas mainly focus on addressing the limitations of current sensor performance and data analysis capabilities [364,365]. These shortcomings can be tackled through approaches such as developing new materials (e.g., nanomaterials), optimizing sensor design, improving surface functionalization technologies, and enhancing energy supply techniques [366,367,368]. By integrating and deeply analyzing multidimensional data, the value of individual data can be maximized, enabling dynamic adjustments to medication regimens to achieve precise personalized treatment goals [369]. Furthermore, it may even become possible to establish precise dosages, and specific medication plans before administration. Looking forward, the development of cardiovascular drug efficacy evaluation will continue to advance toward personalized treatment, laying a foundation for safe, efficient, and individualized therapeutic solutions by accurately monitoring patients’ drug responses and their dynamic changes [292,297,343,370].
